# Polyampholyte Hydrogels in Biomedical Applications

**DOI:** 10.3390/gels3040041

**Published:** 2017-11-04

**Authors:** Stephanie L. Haag, Matthew T. Bernards

**Affiliations:** Department of Chemical & Materials Engineering, University of Idaho, Moscow, ID 83843, USA; haag4885@vandals.uidaho.edu

**Keywords:** polyampholyte hydrogels, nonfouling, multi-functional

## Abstract

Polyampholytes are a class of polymers made up of positively and negatively charged monomer subunits. Polyampholytes offer a unique tunable set of properties driven by the interactions between the charged monomer subunits. Some tunable properties of polyampholytes include mechanical properties, nonfouling characteristics, swelling due to changes in pH or salt concentration, and drug delivery capability. These characteristics lend themselves to multiple biomedical applications, and this review paper will summarize applications of polyampholyte polymers demonstrated over the last five years in tissue engineering, cryopreservation and drug delivery.

## 1. Introduction

A significant amount of research is being done with polyampholyte polymers in the biomedical community. Polyampholytes are polymeric systems comprised of both positively and negatively charged monomer subunits. Through the selection of monomers, one can build a polyampholyte with desired properties, tuned to specific biomedical applications. Our previous work evaluated much of the relevant literature prior to 2013 [1,2], so this paper is focused on advances over the past five years. We will first give a brief review of general polyampholyte characteristics with references to more thorough summaries, a discussion of the tunability of these systems, and an evaluation of recent findings using polyampholytes in tissue engineering, cryopreservation applications, and drug delivery.

## 2. General Polyampholyte Characteristics

A detailed explanation of the synthesis and properties of polyampholytes is beyond the scope of this paper and will not be provided here [3,4,5,6]. However, we will give a brief overview of the general characteristics that make polyampholytes attractive for biomedical applications. As mentioned above, polyampholytes contain both anionic and cationic functional groups. The strengths of these functional groups are often divided into four categories, that include both weak anionic and cationic groups, weak anionic and strong cationic groups, strong anionic and weak cationic groups, and lastly, both strong anionic and cationic groups. Table 1 shows the most commonly used monomers based on a survey of the recent literature. It should be noted that Table 1 is focused on summarizing synthetic organic monomer subunits. There is also a range of literature focused on naturally occurring materials that have been modified to include charged functional groups, like chitosan [7,8].

**Figure 1:**
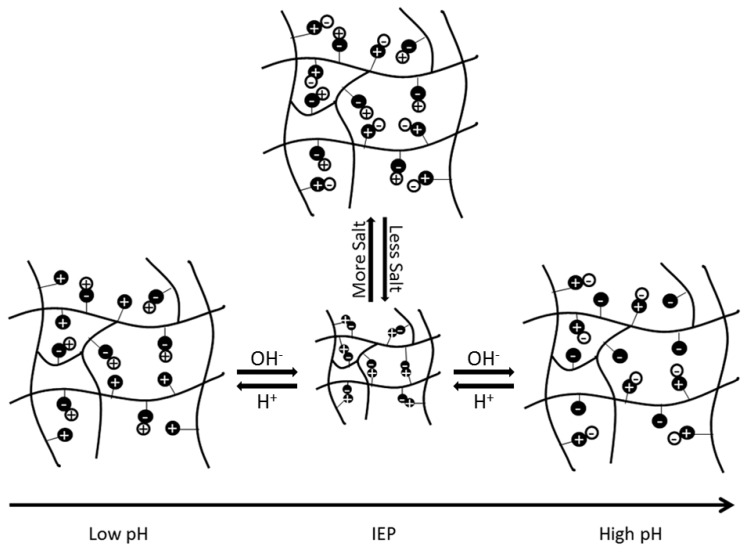
Schematic representation of the impact that changes in pH and salt concentrations have on electrostatic interactions within a polyampholyte hydrogel. This figure is reprinted from Ref. [1] with permission. Copyright 2013, Wiley Periodicals, Inc.

Based on the selection of the underlying functional groups, polyampholytes have a tunable isoelectric point (IEP). The IEP occurs at the pH level when a polyampholyte is overall neutrally charged. The IEP is also the state at which a polyampholyte will have the most compact conformation, due to electrostatic attractions between the balanced, oppositely charged functional groups. As pH increases or decreases from the IEP, the overall charge of the polyampholyte will move further from neutral, causing electrostatic repulsive forces between like-charged regions, to increase and expand the polyampholyte. Similarly, when salt ions are present, the ions disrupt the electrostatic interactions between oppositely charged regions of the subunits. This also causes the polyampholyte to swell, as depicted schematically in Figure 1 [1]. The extent of swelling from pH or salt is ultimately dependent on the composition and architecture of the polymer [1,6]. However, manipulation of these unique electrostatic interactions and system responses has spurred investigation into using these materials in biomedical applications, as detailed throughout the rest of this review. 

Another important general feature of overall charge neutral polyampholyte polymers is their natural nonfouling properties. It has been widely demonstrated [9,10,11,12] and reviewed previously [1,2] that this native resistance to nonspecific protein adsorption is the result of the formation of a strong hydration layer due to interactions between the naturally occurring dipole distribution in water and the charged regions of the underlying polyampholyte substrate. This is important because it is believed that this nonfouling property will lead to a reduced foreign body response in the in vivo environment, as seen with related zwitterionic systems. For example, in a previously reviewed paper, it was shown that polymer brushes composed of equimolar mixtures of 2-(acryl-oyloxy)ethyl trimethylammonium chloride (TMA) and 2-carboxyethyl acrylate (CAA) had only 4.3 ± 1.7 ng/cm^2^ of nonspecific protein adsorption from 100% fetal bovine serum [13]. Furthermore, as demonstrated throughout the remainder of this review, pH changes can be used to modify the net neutral charge of polyampholyte systems, adding in a responsive component to the utilization of these polymers in biomedical applications.

## 3. Mechanical Properties

The composition dependent tunability of polyampholyte systems also provides a unique approach for easily controlling the mechanical properties of the biomaterial. To facilitate better tissue regeneration and integration, it is important for an implanted biomaterial to mimic the native properties of the tissues it is supplanting [14,15]. There is, of course, great variability in the mechanical properties of tissues, as properties range from soft and flexible (skin) to strong, with the ability to absorb impact forces (bone). In addition, biomaterials must also have a high water content, to maintain their biocompatibility, and the ability for cells to penetrate into the material. 

Our group demonstrated the easy tunability of polyampholyte hydrogels utilizing various ratios of monomers in three component hydrogels consisting of positively charged 2-(Acryl-oyloxy)ethyl trimethylammonium chloride (TMA), and varying mixtures of negatively charged 2-carboxyethyl acrylate (CAA) and 3-sulfopropyl methacrylate (SA) monomers [16]. Furthermore, the crosslinker density was also used as a mechanism for further tuning the mechanical properties. It was demonstrated that both the density of the crosslinker, as well as the ratio of monomers in the hydrogel, altered the fracture strength and Young’s Modulus. At crosslinker densities of 1× and 2× (1:0.076 and 1:0.152 monomer/crosslinker ratios), the mechanical properties were dependent upon the exact combination of monomer subunits, while at a 4× crosslinker density, the crosslinker became the controlling factor. However, this study clearly demonstrated the easily tuned mechanical properties of polyampholyte systems with low crosslinker densities. In a similar fashion, Jian and Matsumura were able to controllably tune the mechanical properties of their nanocomposite hydrogel designed with carboxylated poly-l-lysine (COOH-PLL), and synthetic clay laponite XLG, by changing the laponite concentration (composition dependence) or the density of the polyethylene glycol with *N*-hydroxy succinimide ester (PEG–NHS) crosslinker [17]. Changing the crosslinker density or monomer concentration are also common tuning mechanisms for mechanical properties [18,19,20,21,22]. 

A great deal of both theoretical and experimental study has been conducted to better understand the fracture mechanisms of polyampholyte gels, for use in guiding the design of stronger or more tunable systems [23]. Above a critical loading stress, moderately chemically crosslinked hydrogels resisted creep flow, while physically crosslinked and lightly chemically crosslinked hydrogels experience creep rupture. However, at large stresses, creep behavior indicated that both physically and chemically crosslinked hydrogels undergo bond breaking mechanisms. These results confirm that chemical bonds are stronger than physical bonds, therefore, chemically crosslinked systems show an improvement over systems with only ionic bonds [24]. However, the incorporation of physical crosslinks has positively influenced fracture behavior of viscoelastic hydrogels through reduced deformation rate [25] and crack blunting [26].

**Figure 2:**
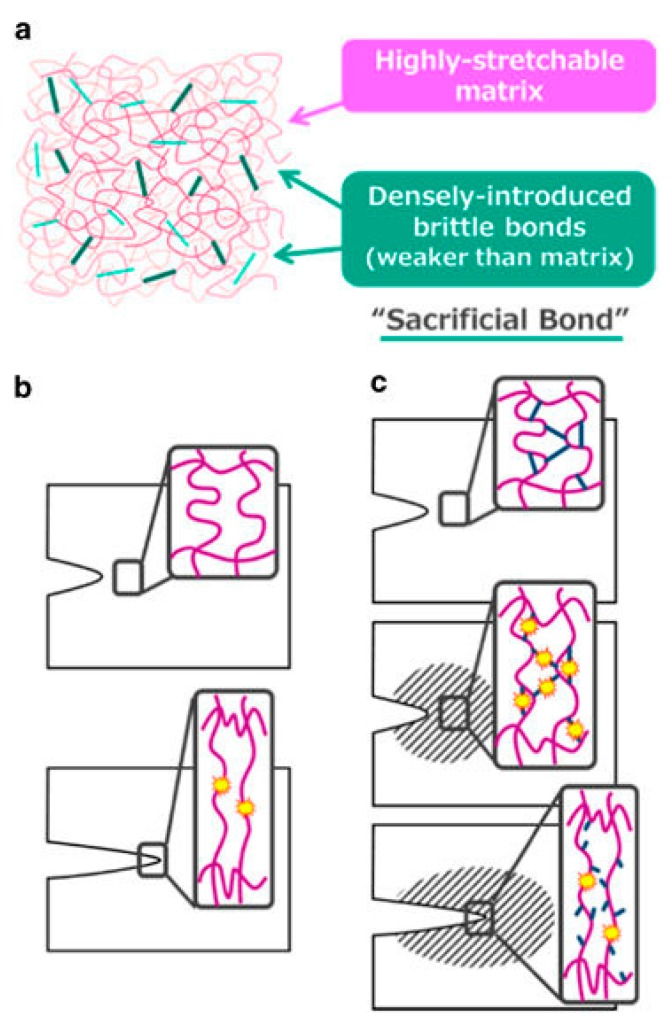
(**a**) General structure of a tough gel based on the sacrificial bond principle consisting of a highly stretchable matrix with a high density of brittle bonds; (**b**) Possible fracture processes of a single network gel; (**c**) Possible fracture processes of a sacrificial bond gel. The brittle bonds are widely ruptured prior to the macroscopic crack propagation around the crack tip (shadowed zone). This figure is reprinted from Ref. [27] with permission. Copyright 2017, the Society of Polymer Science, Japan.

Due to the beneficial features of both chemical and physical crosslinks, recent studies have approached the development of mechanically strong hydrogels by combining the two mechanisms in an approach referred to as the sacrificial bond principle [14,20,27,28,29,30,31,32]. The sacrificial bond principle is based on the formation of a highly stretchable base matrix, with a high density of brittle sacrificial bonds that are weaker than the base matrix. During stress, the brittle bonds break before the stretchable base matrix, leading to improved mechanical performance. Figure 2 shows a schematic of possible fracture processes with and without sacrificial bonds present [27].

These sacrificial bonds can be covalent bonds, hydrogen bonds, ionic bonds, or hydrophobic interactions, depending on the polymer matrix. They can also be incorporated into the base matrix with multiple approaches, including double network gels, ionically linked gels, metal ion chelation, and composite gels [27,33]. The resulting hydrogels from all of these approaches show great mechanical strength, energy dissipation, and force dispersion, to slow down fracture and crack propagation [20]. In just one representative example, the use of a double network hydrogel composed of poly(2-acrylamido-2-methylpropanesulfonic acid) and poly(acrylamide) was shown to improve the compressive fracture stress from 0.4–0.8 to 17.2 MPa [34].

With polyampholyte double network hydrogels showing irreversible deformation, however, efforts started on the use of other types of bonds that could be reversible and self-healing. Some work has been done using electrostatic interactions and hydrophobic interactions. The mechanical properties are extremely dependent on pH, as the interactions that hold the structure together can be weak or strong, depending on the charged state of the monomers. Furthermore, the material will also swell and collapse with changes in pH [28,29]. One study added partially quaternized poly(4-vinylpyridine) into an elastic hydrogel, thereby introducing electrostatic, hydrophobic, and hydrogen bonding interactions to better dissipate energy. This resulted in an increase in fracture energy from 44 to 1000 J/m^2^ [30].

In polyampholytes, it is common to take advantage of the electrostatic interactions as a secondary sacrificial bond, to toughen materials via the presence of oppositely charged functional groups distributed throughout the system. Strong electrostatic interactions act as permanent crosslinks, and weaker interactions reversibly break and reform, which dissipates energy and toughens the gels [14,32]. These bonds can also occur via both inter- and intra-chain interactions. Polyampholytes and polyion complex hydrogels (PIC) both contain oppositely charged functional groups and have potential as tough, self-healing gels. PICs are formed from electrostatic interactions between oppositely charged polyelectrolye polymers upon mixing. Polyampholytes form the toughest hydrogels around zero net charge, where PIC systems can form tough gels at weakly off-balanced charge compositions. PICs are typically tougher than polyampholytes when they have the same monomer compositions, due to the fact that PIC hydrogels form at lower concentrations than polyampholytes [31]. 

Additional approaches have also been used to improve the mechanical properties of hydrogels based on ionic bonding. In one example, the removal of co-ions prior to gelation was shown to facilitate improved ionic bond formation [35]. In another study, Cui et al. developed a method referred to as pre-stretching, where hydrogels are prepared and then stretched. This stretching helps align the chains parallel to each other, as opposed to the original random alignment. When the chains are parallel, stronger ionic bonds form, which in turn strengthens the overall polyampholyte hydrogel [36]. Fang et al. explored a similar approach to attain a tough and stretchable hydrogel by altering the structure of the material [37]. Starting with a protein-based hydrogel, they forced the unfolding of the globular domains. The subsequent collapse and aggregation of the unfolded material allows for physical intertwining and linking through electrostatic interactions. The resulting hydrogels have the unusual properties of a negative swelling ratio, high stretchability, and toughness.

Byette et al. took inspiration from the mechanisms used by mussels to attach to wet surfaces as an approach to toughen polyampholyte materials [38]. Mussels use byssus, a protein-based material, to secure themselves to solid surfaces. Byssus shows a self-healing ability combined with strength, partially due to metal ions forming sacrificial bonds with the amino acid subunits. Byette et al. created a hydrogel from byssus protein hydrolysate, and treated it with Ca^2+^ or Fe^3+^. The films with Fe^3+^ showed the greatest increase in strength and toughness. A similar approach was used by Huang et al., who made a semi-interpenetrating polymer network composed of carboxymethyl chitosan (CMCH), acrylamide, and maleic acid with carboxylic–Fe^3+^ interactions serving as ionic sacrificial bonds [39]. By changing the ratio of maleic acid and the concentration of Fe^3+^, the best hydrogels showed a tensile stress of 1.44 MPa. Additionally, the CMCH provided the gels with antibacterial characteristics against *Staphylococcus aureus* and Gram negative *Escherichia coli*.

## 4. Tissue Engineering Applications

**Figure 3:**
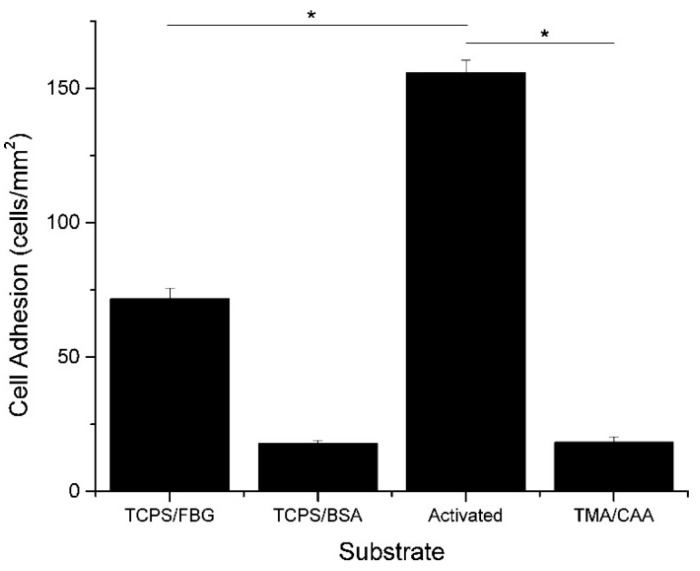
Average number of MC3T3-E1 cells (cells/mm^2^) that adhered to tissue culture polystyrene (TCPS) and TMA/CAA hydrogels with or without adsorbed or conjugated proteins. * Represents a statistically significant difference between the surfaces being compared (*p* < 0.05). This figure is reprinted from Ref. [41] with permission. Copyright 2013, American Chemical Society.

Polyampholyte hydrogels are an attractive option for tissue engineering, due to the general characteristics described above. In addition to their tunable, responsive, and nonfouling properties, they also have a high moisture holding capacity, which is generally associated with biocompatibility. Our group has demonstrated multifunctional polyampholyte hydrogels for tissue engineering using TMA and CAA monomer subunits [40,41]. These gels show excellent resistance to nonspecific protein adsorption, including negatively charged fibrinogen (FBG) and positively charged lysozyme (LYZ), and they prevent the short-term adhesion of MC3T3-E1 cells [41]. The elimination of nonspecific cell adhesion is intended to reduce the occurrence of the foreign body response in the in vivo environment, but it is not desirable for facilitating tissue regeneration through the implanted scaffold. However, the multifunctional capabilities of the polyampholyte hydrogel platform demonstrated in this work provides an easy mechanism for incorporating cell adhesive biological cues. The pH responsive nature of the CAA monomer can be taken advantage of with the use of *N*-(3-dimethylaminopropyl)-*N*′-ethylcarbodiimide hydrochloride/*N*-hydroxysuccinimide (EDC/NHS) bioconjugation chemistry to covalently attach bioactive signaling molecules. This was used to attach FBG, which subsequently facilitated MC3T3-E1 cell adhesion to the hydrogel, as demonstrated in Figure 3 [41]. Furthermore, the background hydrogel (locations without conjugated FBG) was tested, and verified that it retained the native nonfouling properties away from the conjugated proteins, upon return to neutral pH [41]. It is believed that the incorporation of tissue specific biological cues will facilitate targeted cell adhesion and interrogation interactions. This multifunctional capability is not limited to just TMA/CAA polyampholyte hydrogels, either. Three component polymers, using equimolar combinations of positively charged TMA and varying combinations of negatively charged CAA and SA monomers, have also shown the same nonfouling properties and pH dependent protein conjugation capabilities, regardless of the underlying charge balanced composition [16]. This combination of nonfouling properties, protein conjugation capability, and tunable cell adhesion suggests polyampholyte hydrogels have excellent potential for applications as tissue engineering scaffolds.

Advances in the application of polyampholyte hydrogels for tissue engineering are not limited to our efforts. For example, Jian and Matsumura developed a nanocomposite hydrogel using COOH–PLL and synthetic clay laponite XLG, that showed promise as a tissue engineering scaffold due to its controlled release profiles, good mechanical properties, and cell adhesion capability [17]. These gels were cytocompatible, and had adjustable degradation properties. Furthermore, cell adhesion was tunable by controlling the hydrogel formulation. When the polymer chains were covalently cross-linked with PEG-NHS, it hid some of the laponite surface and reduced cell adhesion. Alternatively, when the hydrogels were only physically crosslinked (no PEG–NHS), there was more exposed laponite surface area, leading to enhanced cell attachment. 

## 5. Cryopreservation Applications

Another important aspect of tissue engineering is the preservation of cells over long-term scenarios. This is most generally done using cryopreservation in a liquid nitrogen cell freezer. In order to prevent cell death, a cryoprotective agent (CPA) is typically added to the cell solution prior to freezing. One of the most commonly used CPAs is dimethyl sulfoxide (DMSO), but it shows high cytotoxicity and needs to be removed quickly after thawing. DMSO has also been seen to influence the differentiation of many cell types. The need for a new and more effective CPA has driven research into the use of polyampholytes for cryopreservation. 

**Figure 4:**
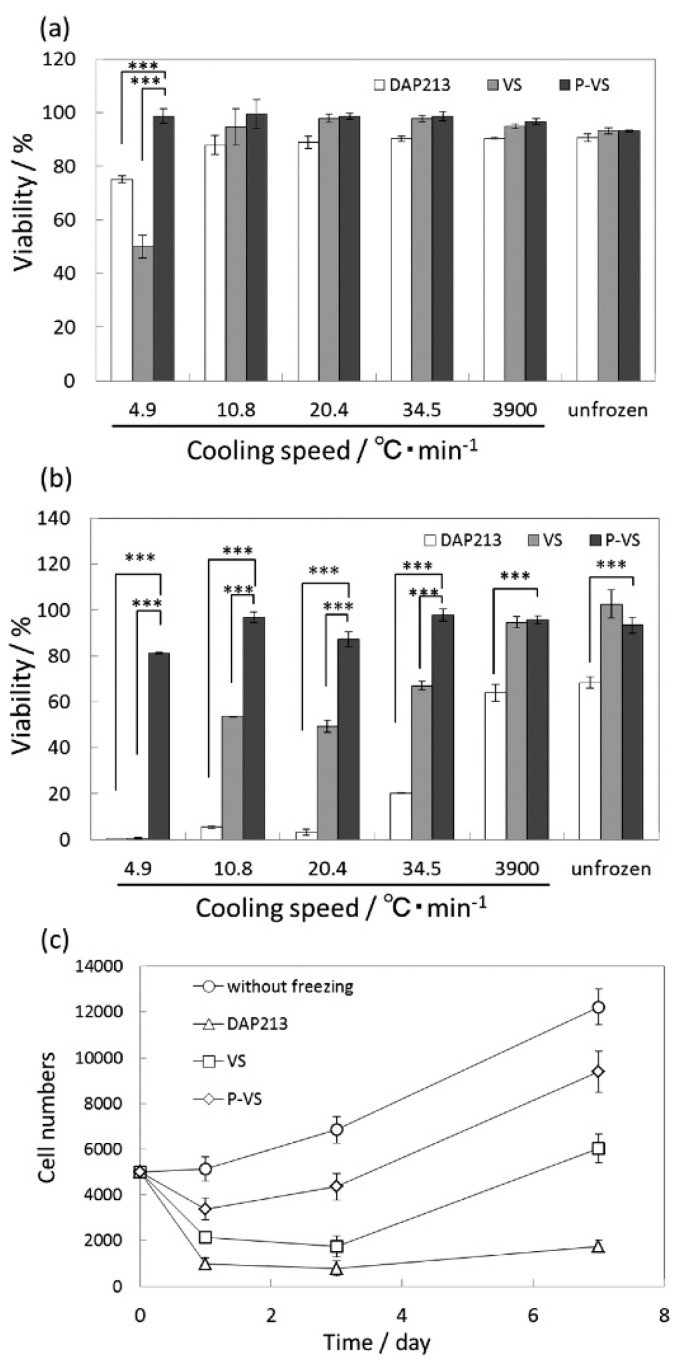
Quantitative viability results of mesenchymal stem cells (MSCs) after slow and fast vitrification with various VSs and different cooling speeds (**a**) immediately after warming and (**b**) after 1 day of culture. (**c**) Cell proliferation curves after slow vitrification at a cooling rate of 10.8 °C/min with various VSs (*** *p* < 0.001). This figure reprinted from Ref. [43] with permission. Copyright 2016, American Chemical Society.

Matsumura et al. demonstrated the use of COOH-PLL as a new polyampholyte CPA for human bone marrow derived mesenchymal stem cells (hBMSCs) [42]. They found that the polyampholyte CPA did not penetrate the cell wall, but instead provided protection by attaching to the membrane. When the ratio of carboxylation was within the range of 0.5–0.8, there was >90% cell viability upon seeding after being frozen for 24 months, with no significant differences compared to cells frozen in the presence of DMSO. The hBMSCs also showed better retention of their properties inherent before freezing, such as differentiation potential, as compared to samples with DMSO as the CPA. COOH-PLL was further tested as a CPA during fast and slow vitrification of two-dimensional cell constructs. Figure 4a,b below, show the cell viability directly after warming, and after one day of culture. It can be clearly seen that there are no significant differences in the cell viability immediately after thawing in the presence of COOH-PLL (denoted as P-VS), DMSO (denoted as DAP213), or no CPA (denoted as VS). After both one day of culture and over longer time periods, the proliferation curves (Figure 4c) show a distinct improvement when the cells were frozen with the polyampholyte CPA, as compared to either DMSO or no CPA. Through these studies, it was concluded that the use of a polyampholyte CPA significantly improved the viability of hBMSCs while maintaining differentiation capacity, making it promising for the long-term storage of tissue engineered constructs [42,43]. 

Based on the positive results seen with COOH-PLL, other polyampholytes have also been investigated as CPAs. These studies were to both expand the formulation range of CPAs, as well as to better understand how polyampholytes protect the cell membrane during freezing. In one example, 2-(dimethylamino) ethyl methacrylate (DMAEMA) and methacrylic acid (MAA) were copolymerized in various ratios [44]. In addition, hydrophobic groups in the form of *N*-butyl methacrylate (Bu-MA) and *N*-octyl methacrylate (Oc-MA) were introduced into the polymer backbone at 2–10% mole percent of the total monomer amount. This range of polyampholyte chemistries were tested, and at an overall solution polymer concentration of 10%, with 5% consisting of Bu-MA or Oc-MA, significantly increased cell viability was seen following freezing. By testing this range of polyampholyte compositions, it was determined that the cryoprotective properties are strongly correlated with hydrophobicity. This approach has also been adapted to the closely related zwitterionic polymers 3-((3-acrylamidoproply)dimethylammonio)-propane-1-sulfonate and 2-((2-methacryloyloxy)ethyl)-dimethylamino acetate [45]. The cryoprotective capabilities of the zwitterionic species were compared to poly(MAA-DMAEMA), and they did not show comparable cell viability, providing further insight into the mechanism of preservation. Through these studies, it was concluded that the cryoprotective property results from strong interactions between the polyampholyte CPA and the cell membrane, which are greatly aided by limited hydrophobic interactions [44,45].

Cell sheets and constructs have added complexity for successful cryopreservation. A dextran-based polyampholyte hydrogel was developed to encapsulate cell constructs prior to cryopreservation, and it has shown promise for tissue engineering applications in preliminary studies [46]. Another variation on the use of COOH-PLL CPAs was explored by Jian and Matsumura. Cells were cryopreserved with 7.5–20% COOH-PLL solutions. After thawing, nanosilicates were injected, turning the solution into a thixotropic hydrogel. Cell viability was excellent, remaining >90% for all tested polyampholyte concentrations. This unique gel system was proposed for direct cell injection for site specific cell delivery and tissue repair, without the need to wash out the cryoprotective agent [47]. Furthermore, the thermoresponsiveness of this class of polyampholyte materials, and their demonstrated biocompatibility, make them promising for other biomaterial and drug delivery applications [48]. 

## 6. Drug Delivery Applications

Due to the naturally occurring responsive nature of polyampholyte polymers addressed earlier, they have gained increasing interest for drug delivery applications. The cryoprotective properties of some polyampholyte formulations, discussed above, have been taken a step further by Ahmed et al. as a novel approach to deliver proteins into cells [49]. Proteins were adsorbed on/into nanoparticles made from hydrophobically modified polyampholytes synthesized by the succinylation of ε-poly-l-lysine with dodecyl succinic anhydride and succinic anhydride. L929 cells were then frozen with the protein loaded nanoparticles as a CPA. The high affinity between the cell membrane and the hydrophobic subunits of the nanoparticles caused the protein-loaded nanoparticles to condense on the peripheral cell membrane during freezing. The adsorbed protein and nanoparticles were found to be internalized after thawing via endocytosis during culture, thereby delivering the protein payload. However, there was a critical concentration above which these nanoparticle delivery systems became cytotoxic. The Matsumura group also adapted this approach to polyampholyte-modified liposomes in additional protein delivery studies, demonstrating its adaptability for protein delivery in immunotherapy applications [50].

At the same time, much of the recent work in polyampholyte mediated drug delivery takes advantage of the pH responsive behavior of polyampholyte systems. For example, chitosan based polyampholytes have recently been shown to have potential in protein delivery applications, as they have exhibited the ability to adsorb and desorb bovine serum albumin (BSA) in a pH dependent manner [7,8]. However, a combination of design characteristics is required to optimize drug delivery that include biocompatibility, multifunctionality, and responsiveness to the microenvironment. Nanogels have been investigated for use as delivery systems, and have shown tremendous promise due to the ability to control drug release, provide the drug protection from degradation, and target specific tissues. Some of the loading and drug release methods include covalent conjugation, passive/diffusion based, or through environmental stimuli, such as pH [51]. 

**Figure 5:**
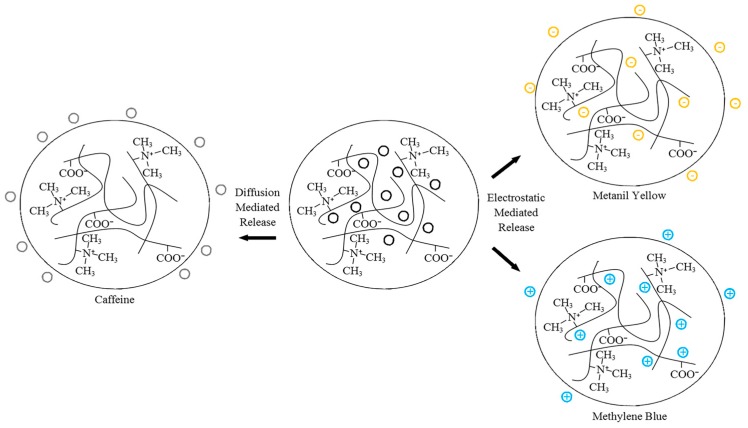
Schematic depicting the release of caffeine, metanil yellow and methylene blue from TMA/CAA gels. This figure is reprinted from Ref. [52] with permission. Copyright 2015, American Chemical Society.

Our group previously investigated the fundamental release characteristics of polyampholyte hydrogels composed of equimolar ratios of TMA and CAA using neutral caffeine, positively charged methylene blue, and negatively charged metanil yellow [52]. These species were selected as methylene blue and metanil yellow have nearly identical molecular weights, thereby eliminating this variable when comparing the release kinetics, while caffeine is approximately one half the size of the other species, to allow for a characterization of the influence of size. Hydrogels were synthesized in the presence of the drug analogues, and then the release characteristics were monitored as a function of crosslinker density, pH, and ionic concentration. The release of the smaller, neutral caffeine molecule was shown to be mediated by diffusion alone, although this release was tunable, based on environmental stimuli induced swelling of the polyampholyte hydrogels. Conversely, the release of the charged molecules was strongly dependent on electrostatic interactions throughout the system, which could be modified through the environmental cues of pH and ionic strength. Figure 5 shows a schematic of the relative drug release levels from the TMA/CAA hydrogel. Importantly, it was also demonstrated that following the release of the various drug molecules, it was verified that the TMA/CAA platforms retained their native nonfouling characteristics. Therefore, this platform shows great potential for long-term biomolecule delivery.

Kudaibergenov et al. also used a variety of guest molecules to characterize the adsorption and release from a macroporous amphoteric cryogel composed of *N*,*N*-dimethylaminoethyl methacrylate and methacrylic acid with a *N*,*N*′-methylenebisacrylamide crosslinker [53]. The guest species tested included methylene blue, methyl orange, sodium dodecylbenzene sulfonate (SDBS), and lysozyme. Lysozyme and methylene blue were adsorbed at pH 9.5, and SDBS and methyl orange were adsorbed at pH 7.5. Similar to the work by Barcellona et al., the binding interactions between the cryogel and the guest molecules was driven by electrostatic forces. However, at the IEP of pH 7.1, the amphoteric cryogel allowed for the release of 93–98% of the adsorbed species. The conclusions drawn by both Barcellona et al. and Kudaibergenov et al. are also supported by simulation based studies that concluded that electrostatic interactions play the most significant role in mediating drug release from polyampholyte systems [54]. 

A variety of specific drug species have also been used to test drug delivery from assorted polyampholyte mediums. Mishra et al. used poly 3-[(methacryoylamino)propyl trimethylammonium chloride-*co*-methacrylic acid] (PMAPTACMAAc) copolymers with various concentrations of monomers and loaded indomethacin (IND) [55]. IND is a nonsteroidal anti-inflammatory drug that is used for the treatment of rheumatoid arthritis, ankylosing spondylitis, and osteoarthritis, to name a few. The hydrogel composition played a large role in the sustained release of IND, and PMAPTACMMAc-5 led to the highest percentage of IND release. This formulation released 75% of the entrapped IND within 8 h and 82% after 12 h. Other hydrogel formulations showed release percentages ranging from ~44% to 77% after 12 h. The release was primarily diffusion based, and it followed non-Fickian release kinetics. Although diffusion is often effective for drug delivery, a controlled release response can provide a more targeted delivery. Salicylic acid was used as a model drug in a polyampholyte composed of casein and poly(*N*-isoproplyacrylamide), and the release was affected by temperature, pH, and crosslinker density [56]. This led Cao et al. to conclude this delivery vehicle was appropriate for orally administered drug delivery. Finally, Sankar et al. demonstrated the pH sensitive release of promethazine hydrochloride from polyampholyte hydrogels containing carbon nanotubes [57]. These nanotubes were incorporated into the hydrogel as an approach to reinforce the mechanical properties of this delivery system, without impacting the drug delivery capabilities.

**Figure 6:**
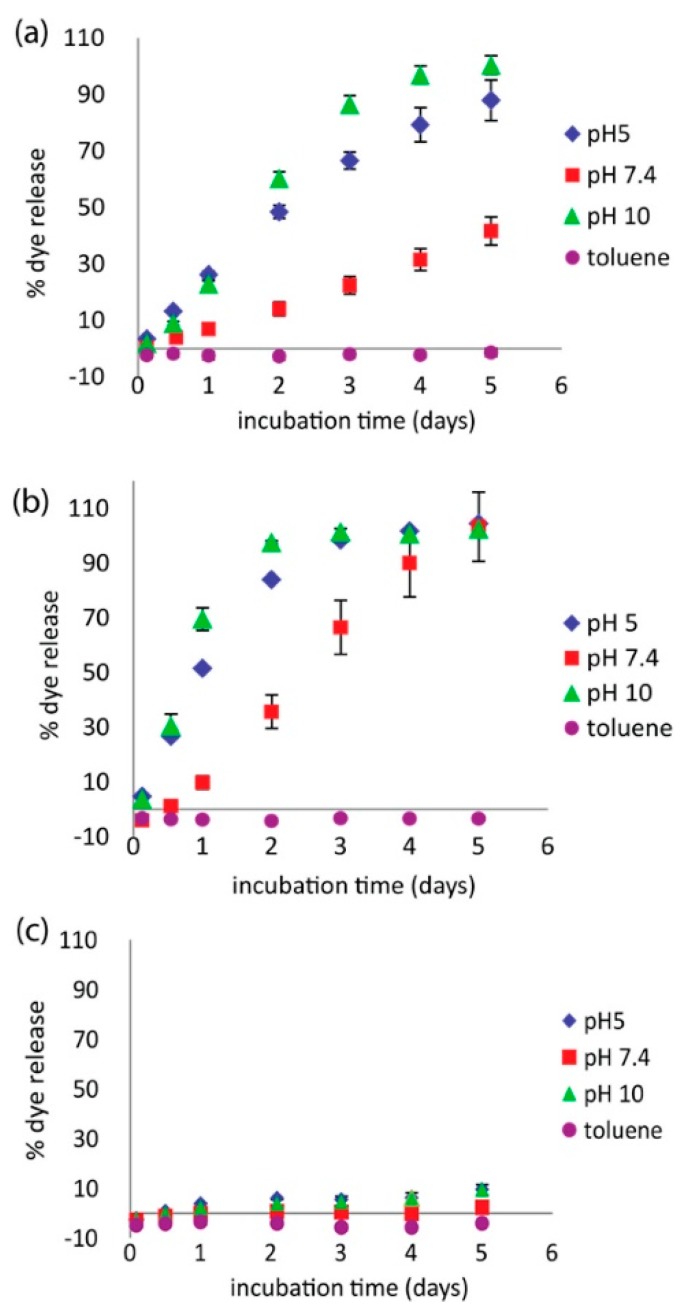
Release profiles of coumarin 1 dye under different solvent conditions for PDDC capsules with (**a**) 3:1 triazine/DETA and (**b**) 1:1 triazine/DETA. (**c**) Control experiments: 1:1 triazine/DETA with TC. This figure is reprinted from Ref. [58] with permission. Copyright 2017, American Chemical Society.

Investigators have also begun incorporating polyampholytes into multicomponent systems to enhance performance or offer additional benefits. For example, Wang et al. examined a polyampholyte hydrogel release system based on pyromellitic diester diacid chloride (PDDC) combined with combinations of diethylenetriamine (DETA) and triazine [58]. This polyampholyte system showed a pH dependent release capability that overcame previous issues seen with related encapsulants formed with terephthaloyl chloride (TC) in place of PDDC. This new microcapsule formulation showed high loading capacity, and steady, controlled release at pH 7.4. It also demonstrated accelerated release at both pH 5 and pH 10, as shown in Figure 6. The release characteristics were also tunable by varying the ratio of DETA to triazine, indicating the ability to refine this microcapsule formulation for tunable release rate applications.

Others has also incorporated polyampholyte polymers into their drug delivery vehicles to add pH responsive release characteristics. For example, Schulze et al. saw potential in lamellar liquid crystalline systems, but the structure did not react to environmental stimuli such as pH. When the polyampholyte poly(*N*,*N*′-diallyl-*N*,*N*′-dimethyl-almaleamic carboxylate) (PalH) was integrated into a lamellar liquid crystalline system of sodium dodecyl sulfate, decanol, and water, it was found that release from the new structure could be tuned by varying the pH or temperature. This suggests it has promise as a new structural material for drug delivery systems [59]. In another example, papacetamol, an analgesic drug, was released from a polyampholyte hydrogel matrix composed of laponite, polyacrylamide and poly(3-acrylamidopropyl) trimethylammonium chloride. Drug release was tested as a function of environmental changes in pH and ionic strength, and in the presence of an electric field. Without an electric field, papacetamol was only released at pH 1.1, but with the application of an electric field, sustained drug release occurred at other pH values [60]. Finally, Ali et al. created a novel polymer containing residues of alendronic acid, that showed pH sensitive responses that were proposed to be used as a drug delivery system [61].

Asayama et al. also incorporated a polyampholyte polymer, carboxymethyl poly(1-vinylimidazole) (CM-PVIm), into an existing system. CM-PVIm was used to coat poly(ethylenimine)/DNA (PEI/DNA) complexes, to reduce nonspecific protein adsorption to this delivery platform. The results demonstrated this coating did not significantly reduce gene transfection or cell viability. Therefore, the authors concluded that CM-PVIm is an effective coating for improved circulation of gene therapy agents [62].

Another application of adding polyampholytes to drug delivery vehicles is based on their strong water holding capacity. Polyampholyte acrylic latexes were incorporated into drug tablet coatings to minimize the amount of water removed from the drugs during the tablet drying step [63]. During the optimization of this approach, Ladika et al. focused on finding a polymer solution with similar viscosity to the industry standard, that contained a much higher concentration of solids. Typical tablet coatings on the market today range from 4–10 wt % solids and the new polyampholyte acrylic latexes showed a range of 37–39 wt % solids. Three types of latexes were explored: weak acid/strong base latexes, strong acid/weak base latexes, and combinations of anionic and cationic latexes. Latex formulations for all three combinations were determined that had viscosities similar to current coating solutions, had higher solids composition, and were pH-tunable, to enable targeted delivery of active pharmaceutical ingredients.

## 7. Future Directions

Throughout this review, many exciting advancements applying polyampholyte hydrogels to biomedical applications were highlighted. However, despite this progress and the clearly demonstrated capabilities of polyampholytes, these materials have not yet been investigated in the in vivo environment in depth. This is the critical next step in the continued development of these materials, and our group is pursuing these efforts in the application of polyampholyte hydrogels for bone tissue engineering. Additionally, while the tunability and responsive properties of polyampholytes have been widely demonstrated, the ease of tuning polyampholyte materials for targeted applications of these capabilities must also be further pursued.

## Figures and Tables

**Table 1:** Common Monomers Used in Polyampholyte Hydrogels.

Chemical Name	Acronym	Monomer Formula	Strength of Functional Group
**Acrylamide**	AM	CH_2_=CHCONH_2_	Weak cation
***N*-[3-(Dimethylamino)propyl] acrylamide**	DMAPAA	CH_2_=CHCONH(CH_2_)_3_N(CH_3_)_2_	Weak cation
**2-(Dimethylamino)ethyl methacrylate**	DMAEM	CH_2_=C(CH_3_)COOCH_2_CH_2_N(CH_3_)_2_	Weak cation
**2-(Diethylamino)ethyl methacrylate**	DEAEM	CH_2_=C(CH_3_)CO_2_CH_2_CH_2_N(C_2_H_5_)_2_	Weak cation
**[2-(Methacryloyloxy)ethyl] trimethylammonium chloride**	TM	CH_2_=C(CH_3_)CO_2_CH_2_CH_2_N(CH_3_)_3_Cl	Strong cation
**2-(Acryloyloxy ethyl)trimethyl ammonium chloride**	TMA	CH_2_=CHCO_2_CH_2_CH_2_N(CH_3_)_3_Cl	Strong cation
**[3-(Methacryloylamino)propyl] trimethylammonium chloride**	MAPTAC	CH_2_=C(CH_3_)CONH(CH_2_)_3_N(CH_3_)_3_Cl	Strong cation
**2-Carboxyethyl acrylate**	CAA	CH_2_=CHCO_2_(CH_2_)_2_CO_2_H	Weak anion
**Methacrylic acid**	MAA	CH_2_=C(CH_3_)COOH	Weak anion
**Acrylic acid**	AA	CH_2_=CHCOOH	Weak anion
**Carboxylated poly-l-lysine**	COOH-PLL	NH_2_(CH_2_)_4_CHNH_2_COOH	Weak anion
**3-Sulfopropyl methacrylate potassium salt**	SA	H_2_C=C(CH_3_)CO_2_(CH_2_)_3_SO_3_K	Strong anion
**2-Sulfoethyl methacrylate**	SE	H_2_C=C(CH_3_)CO_2_(CH_2_)_2_SO_3_H	Strong anion
